# [Retracted] tRNA-derived fragment tRF-Glu49 inhibits cell proliferation, migration and invasion in cervical cancer by targeting FGL1

**DOI:** 10.3892/ol.2025.15150

**Published:** 2025-06-23

**Authors:** Yang Wang, Wenying Xia, Fangrong Shen, Jinhua Zhou, Yanzheng Gu, Youguo Chen

Oncol Lett 24: 334, 2022; DOI: 10.3892/ol.2022.13455

Following the publication of this paper, it was drawn to the Editor's attention by a concerned reader that the tissue microarray data shown in Fig. 2A, and one of the Transwell cell migration assay data panels shown in Fig. 3D were strikingly similar to data that had already appeared in a pair of previously published articles written by a different research group at different research institutes.

Owing to the fact that the contentious data in the above article had already been published prior to its submission to *Oncology Letters*, the Editor has decided that this paper should be retracted from the Journal. The authors were asked for an explanation to account for these concerns, but the Editorial Office did not receive a reply. The Editor apologizes to the readership for any inconvenience caused.

